# Impact of interleukin‐1β single nucleotide polymorphisms and depressive symptoms in individuals with chronic viral hepatitis

**DOI:** 10.1002/kjm2.12776

**Published:** 2023-11-08

**Authors:** Hsin‐Yi Huang, Rwei‐Ling Yu, Wei‐Fang Tsai, Wan‐Long Chuang, Jee‐Fu Huang, Chia‐Yen Dai, Chun‐Hsiang Tan

**Affiliations:** ^1^ Department of General Medicine Kaohsiung Medical University Hospital, Kaohsiung Medical University Kaohsiung Taiwan; ^2^ Institute of Behavioral Medicine, College of Medicine National Cheng Kung University Tainan Taiwan; ^3^ Graduate Institute of Clinical Medicine, College of Medicine Kaohsiung Medical University Kaohsiung Taiwan; ^4^ Department of Internal Medicine and Hepatitis Center Kaohsiung Medical University Hospital, Kaohsiung Medical University Kaohsiung Taiwan; ^5^ Department of Neurology Kaohsiung Medical University Hospital, Kaohsiung Medical University Kaohsiung Taiwan

**Keywords:** Beck Depression Inventory, depressive symptoms, hepatitis B, hepatitis C, interleukin‐1β

## Abstract

Elevated levels of interleukin 1β (IL‐1β) have been identified in patients with chronic viral hepatitis and have been associated with depressive symptoms. Given the high prevalence of depression in this patient population, this study sought to explore the potential influence of IL‐1β genetic variations on the severity of depressive symptoms. In a cohort of 181 Taiwanese patients with chronic viral hepatitis, we investigated the impact of five common IL‐1β single nucleotide polymorphisms (SNPs), including rs16944, rs1143627, rs1143630, rs1143643, and rs3136558, on depressive symptoms using the Beck's Depression Inventory‐II. Additionally, we analyzed the primary domains of IL‐1β‐related depressive symptoms according to Beck's six symptom categories of depression. Our analysis revealed significant associations between depressive symptoms and three intronic IL‐1β SNPs. After controlling for age, sex, marital status, and education level, patients with the rs1143630 GG, rs1143643 CC, and rs3136558 AA genotypes demonstrated higher severity of depressive symptoms in the domains of indecision (*p* = 0.004), agitation (*p* = 0.001), and feelings of punishment (*p* = 0.005), respectively, compared to rs1143630 GA+AA, rs1143643 CT, and rs3136558 AG+GG genotypes. According to Beck's categorization, these symptoms can be classified into three dimensions: disturbances in emotion regulation, energy, and cognition. Our findings demonstrate the association between IL‐1β polymorphisms and depressive symptoms and suggest a potential underlying mechanism for specific depressive symptoms within the chronic viral hepatitis population. These insights could improve our understanding and treatment of depressive symptoms in individuals with viral hepatitis.

**Abbreviations**
BDI‐II: Beck Depression Inventory‐II
C‐BDI‐II: Chinese version of the Beck Depression Inventory‐II
CNS: central nervous system
DSM‐IV: Diagnostic and Statistical Manual of Mental Disorders‐IV
HBV: Hepatitis B virus
HCV: Hepatitis C virus
IL‐1β: Interleukin‐1β
LD: linkage disequilibrium
MAF: minor allele frequency
NLRP3: NLR family pyrin domain‐containing 3
SNP: single nucleotide polymorphism

## 1 INTRODUCTION

Individuals with chronic viral hepatitis frequently exhibit depressive symptoms^1^ and have elevated levels of pro‐inflammatory cytokines, such as tumor necrosis factor‐α,^2^, ^3^ interleukin‐1β (IL‐1β),^3^ and interleukin‐6,^2^ compared to their healthy counterparts.^3^ The association between the cytokine levels and the severity of depressive symptoms suggests elevated cytokines as unique pathogenic mechanisms underlying depression induced by viral hepatitis. Chronic viral infections result in increased pro‐inflammatory cytokines capable of penetrating the blood–brain barrier and augmenting cytokine secretion by cerebral microglial cells.^4^ These signals modulate brain activity through various pathways linked to depression, including alterations in neurotransmitter metabolism, neuroendocrine function, and neural plasticity.^4^, ^5^

Among the various pro‐inflammatory cytokines released during chronic viral hepatitis infection, emerging evidence suggests that IL‐1β may play a pivotal role in the pathogenesis of depression.^6^ Secreted by activated macrophages, IL‐1β can activate, proliferate, and differentiate immune cells. The widespread expression of IL‐1β receptor on numerous cell types, including immune cells, endothelial cells, and CNS cells, also suggests the profound impact of IL‐1β.^7^ Previous research has suggested several single nucleotide polymorphisms (SNPs) of the IL‐1β gene to be implicated in depression, such as rs16944, rs1143627, and rs1143643.^8^ However, despite extensive research endeavors investigating the association between IL‐1β gene polymorphisms and depression,^9^, ^10^, ^11^, ^12^, ^13^, ^14^, ^15^, ^16^, ^17^ existing studies have yielded inconsistent results.^9^, ^10^, ^11^, ^12^, ^13^

Therefore, this study aims to explore the potential influence of IL‐1β on depressive symptoms in chronic viral hepatitis patients by examining whether IL‐1β SNP genotypes affect depressive symptom phenotypes.

## 2 METHODS

### 2.1 Participants

A total of 181 participants with chronic hepatitis B virus (HBV), chronic hepatitis C virus (HCV), or both, were recruited from Kaohsiung Medical University Hospital between 2019 and 2021, and all participants were of Eastern Asian ethnicity. The inclusion criteria for participants with chronic HBV infection were a positive hepatitis B surface antigen test lasting over 6 months. For those with chronic HCV, criteria included a positive anti‐HCV immunoglobulin test and a history of HCV presence, confirmed via polymerase chain reactions, for over 6 months. The patients’ demographic information, including age, sex, and hepatitis status, was obtained from medical records. Baseline characteristics, such as educational attainment, marital status, and past medical history (including malignancy, psychiatric disorders, and chronic kidney disease), were gathered through interviews. The management of HBV and HCV in participants followed EASL 2017^18^ and AASLD‐IDSA guidelines,^19^ respectively.

Exclusion criteria included patients under 50 years of age, and those with a history of malignancy, severe psychiatric disorders, acute infection, or chronic kidney disease (stage IV or V). In accordance with the Helsinki Declaration's ethical principles, all participants provided written informed consent. The study procedures were approved by the ethical research committee of Kaohsiung Medical University Hospital.

### 2.2 Assessment of liver fibrosis

The information on the Metavir score was available in 33 of the 181 participants (18.2%). Thus, we adopted the FIB‐4 score to measure fibrosis staging. The FIB‐4 score is a noninvasive scoring system based on the patient's age, platelet count, aspartate transaminase, and alanine transaminase. These were obtained within a 6‐month period from the date of the assessment of depressive symptoms.

### 2.3 Assessment of depressive symptoms

Depressive symptoms were evaluated using the Chinese BDI‐II (C‐BDI‐II) questionnaire.^20^ The C‐BDI‐II is a self‐report instrument consisting of 21 items that has been frequently employed as an indicator of depression severity with strong reliability and validity.^21^ The C‐BDI‐II employs a four‐point (0–3) scale for its 21 items, with 0 indicating “no symptoms” and 3 indicating “severe symptoms.” Overall scores range from 0 to 63 points, with higher scores reflecting more severe depressive symptoms. Participants responded to the C‐BDI‐II based on their daily situation within the 2 weeks preceding the assessment. Due to Chinese social customs, the data for the 21st item (“loss of interest in sex”) was deemed potentially inaccurate and excluded from further statistical analysis to prevent bias, as almost a quarter of the participants did not provide an answer. For classification using BDI‐II items,^22^ Beck's categorization divided items into six main dimensions^23^: regulation of emotion (items 1, 4, 10, 13), motivation (items 2, 9), focused attention (items 12, 19), cognitive distortions (items 3, 5, 6, 7,8, 14), energy regulation (items 11, 15, 17, 20), and physiological/vegetative (items 16, 18, 21).

### 2.4 Genotyping and linkage disequilibrium evaluation

Genomic DNA was extracted from participants’ peripheral blood leukocytes using the Genomic DNA Extraction Kit (Geneaid, Taiwan). The IL‐1β SNPs were determined with the C2‐58 Axiom Genome‐Wide TWB 2.0 Array Plate on the Affymetrix GeneChip platform. The genotyping results were used for constructing the pairwise linkage disequilibrium (LD) pattern among IL‐1β genetic variants. HaploView 4.2 software (https://haploview.software.informer.com/) was employed for LD mapping.

### 2.5 Statistical analysis

Qualitative variables were represented as numbers or percentages, while quantitative variables were presented as means ± standard deviations. χ^2^ tests were employed for analyzing qualitative parameters, and the Kolmogorov–Smirnov test was utilized to assess the normality of quantitative parameters. Quantitative parametric variables were analyzed using the Student's *t*‐test or one‐way ANOVA, while nonparametric quantitative analyses were performed using the Mann–Whitney *U*‐test or Kruskal–Wallis *H*‐test. Confounding variables (age, sex, marital status, and education attainment) were controlled using Quade's test. To avoid a type II error resulting from insufficient statistical power due to a small sample size, we combined the group with homozygous genotypes that had fewer than 30 cases with the group possessing heterozygous genotypes. This allowed us to achieve a case number of over 30 in each study group.

All statistical analyses were performed using IBM SPSS Statistics, Version 22.0 (IBM Corp., Armonk, NY). With the Bonferroni correction, the significance level was set at *p* < 0.0125 (four selected IL‐1β SNPs were analyzed, so *p* = 0.05/4 = 0.0125).

## 3 RESULTS

### 3.1 Study participants

This study examined the association between IL‐1β genotypes and depressive symptoms in 181 viral hepatitis patients, including 96 females and 85 males. The participants comprised 67 individuals with chronic HBV, 104 with chronic HCV, and 10 with both. Among those with chronic HCV, 80 achieved sustained virologic response (SVR), defined as the absence of HCV RNA by polymerase chain reaction 6 months post anti‐HCV therapy. The average age of the participants was 61.99 ± 7.46 years. Of the 77 participants with either HBV alone or both HBV and HCV, 60 received antiviral treatment for HBV. Details regarding HCV treatment, HCV genotypes, fibrosis staging, and the association between HCV genotypes and depressive symptoms are provided in Tables S1, S2, S3, and S4, respectively (Data S1). The mean value of FIB‐4 score of all participants was 2.66 ± 6.01.

### 3.2 Genotype distributions

**FIGURE 1:**
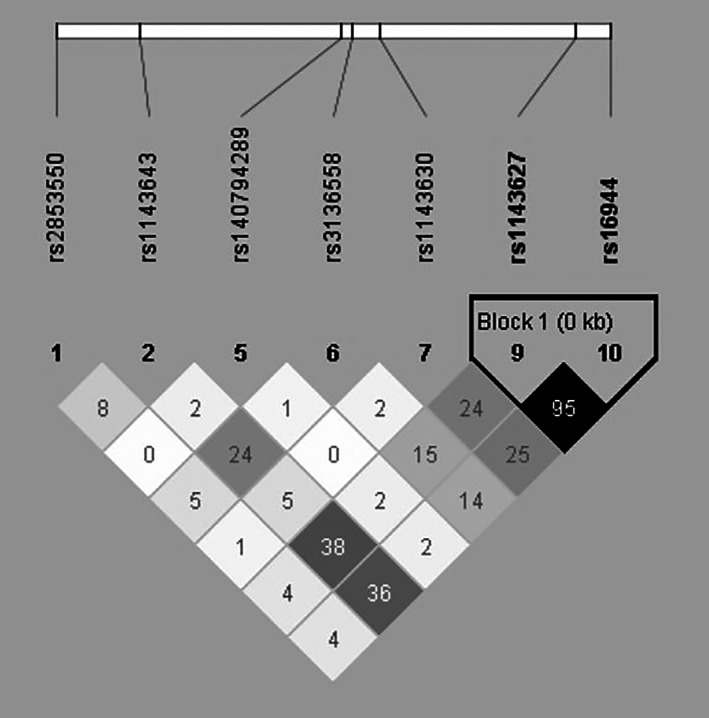
Haploview analysis of linkage disequilibrium among IL‐1β SNPs. The pairwise *r* ^2^ measures of linkage disequilibrium were calculated using Haploview software v4.2, based on unrelated Taiwanese individuals. *R* ^2^ values are displayed in white for *r* ^2^ < 15%, with shades of gray indicating intermediate values (15% < *r* ^2^ < 80%), and black representing high values (*r* ^2^ > 80%). The squares contain the respective *r* ^2^ scores for pairwise linkage disequilibrium. A single block (distance <1 kb) was identified among IL‐1β SNPs, with a high level of LD observed between rs16944 and rs1143627 (D′ = 1.0, *r* ^2^ = 0.957, *p* < 0.00001). The *r* ^2^ value of 0.957 between rs16944 and rs1143627 signifies complete linkage disequilibrium in a reverse pattern (rs16944 A/rs1143627 G or rs16944 G/rs1143627 A).

We conducted an investigation of 10 IL‐1β SNPs, as shown in Table 1, and selected five SNPs with a minor allele frequency (MAF) exceeding 10% for further analysis for adequate sample size for each genotype. The genetic distribution of the selected SNPs was found to conform to the Hardy–Weinberg equilibrium (Table 1). Pairwise LD was computed among the seven IL‐1β SNPs that have a non‐zero MAF, and we observed the presence of one block (distance <1 kb) among them, as well as high LD between rs16944 and rs1143627 (*D*′ = 1.0, *r* ^2^ = 0.957, *p* < 0.00001), as illustrated in Figure 1. The *r* ^2^ value of 0.957 between rs16944 and rs1143627 indicates strong LD between these SNPs. Therefore, we chose rs1143627, the SNP with a higher MAF, for association analysis.

**TABLE 1:** Description of IL‐1B SNPs and allele frequency.

SNP ID	Position	Role	Minor/major allele	MAF	HWE (*p*‐value)
rs16944	113,594,867	Promoter	A/G	46.7%	χ^2^ = 3.215 (*p* = 0.200)
rs1143627	113,594,387	Promoter	G/A	47.8%	χ^2^ = 2.221 (*p* = 0.329)
rs1143630	113,591,655	Intron	T/G	18.5%	χ^2^ = 3.072 (*p* = 0.215)
rs1143636	112,831,797	Intron	G/A	0%	
rs1143642	112,830,976	Intron	A/G	0%	
rs1143643	113,588,302	Intron	C/T	48.9%	χ^2^ = 0.205 (*p* = 0.902)
rs2853550	112,829,544	Downstream	A/G	7.7%	χ^2^ = 0.491 (*p* = 0.782)
rs3136558	113,591,275	Intron	G/A	39.5%	χ^2^ = 4.745 (*p* = 0.093)
rs3917350	112,836,120	Intron	G/A	0%	
rs140794289	112,833,537	Synonymous variant	A/G	2.8%	χ^2^ = 0.094 (*p* = 0.954)

Abbreviations: HWE, Hardy–Weinberg equilibrium; MAF, minor allele frequency; SNP, single nucleotide polymorphism.

### 3.3 IL‐1β SNP rs1143627 is not significantly associated with depressive symptoms

The frequency of the rs1143627 minor G alleles was 47.8% (Table 1). Demographic characteristics were stratified based on the genotypes of the IL‐1β SNPs rs1143627. Among the three groups, no significant differences were observed in age, sex, education, marital status, proportions of individuals achieving SVR, HBV/HCV infection rates, FIB‐4 scores, or HCV genotypes (Table 2). We found no significant differences in any of the BDI score items (Table 2). Even after controlling for potential confounding factors such as age, sex, education, and marital status using Quade's test, the absence of significant differences persisted. These findings suggest that the rs1143627 SNP does not have a significant impact on the severity of depressive symptoms.

**TABLE 2:** Clinical characteristics and BDI‐II scale results stratified by rs1143627 genotypes.

Variables	GG (*n* = 36)	AG (*n* = 101)	AA (*n* = 44)	Statistic, *p*‐value	Quade's test
Age (years)	60.58 ± 6.030	62.23 ± 7.987	62.61 ± 7.298	*F* = 0.842, *p* = 0.433	
Male/female	13 (36.1%)/23 (63.9%)	47 (46.5%)/54 (46.5%)	25 (46.8%)/19 (43.2%)	χ^2^ = 3.425, *p* = 0.180	
Education (years)	11.89 ± 4.139	11.67 ± 3.085	11.43 ± 3.546	*F* = 0.144, *p* = 0.866	
Marital status (single/married/divorced/widowed)	2/31/1/2	7/84/2/8	4/39/0/1	χ^2^ = 3.172, *p* = 0.787	
Sustained virologic response	15 (55.6%)	46 (74.2%)	19 (76.0%)	χ^2^ = 3.641, *p* = 0.162	
FIB‐4 score	2.18 ± 1.130	3.07 ± 7.976	2.11 ± 1.200	*F* = 0.535, *p* = 0.587	
HCV/HBV/both	23/9/4	59/39/3	22/19/3	χ^2^ = 6.171, *p* = 0.168	
HCV genotype (1a, 1b, 2, 1b/2, 1b/3, 2/4) (number)	0, 11, 13, 1, 0, 0	2, 23, 24, 1, 0, 0	0, 7, 14, 0, 1, 1	χ^2^ = 9.712, *p* = 0.375	
BDI‐1: Sadness	0.06 ± 0.232	0.03 ± 0.222	0.02 ± 0.151	*H* = 1.277, *p* = 0.528	*F* = 0.390, *p* = 0.678
BDI‐2: Pessimism	0.14 ± 0.424	0.21 ± 0.553	0.30 ± 0.701	*H* = 0.913, *p* = 0.634	*F* = 0.366, *p* = 0.694
BDI‐3: Past failure	0.14 ± 0.487	0.15 ± 0.498	0.18 ± 0.540	*H* = 0.261, *p* = 0.878	*F* = 0.224, *p* = 0.799
BDI‐4: Loss of pleasure	0.06 ± 0.232	0.09 ± 0.286	0.11 ± 0.387	*H* = 0.454, *p* = 0.797	*F* = 0.418, *p* = 0.659
BDI‐5: Feeling of guilty	0.14 ± 0.351	0.13 ± 0.337	0.20 ± 0.408	*H* = 1.418, *p* = 0.492	*F* = 1.065, *p* = 0.347
BDI‐6: Feelings of being punished	0.19 ± 0.710	0.12 ± 0.612	0.18 ± 0.657	*H* = 0.056, *p* = 0.973	*F* = 0.076, *p* = 0.927
BDI‐7: Self‐dislike	0.06 ± 0.232	0.05 ± 0.260	0.09 ± 0.362	*H* = 0.564, *p* = 0.754	*F* = 0.328, *p* = 0.721
BDI‐8: Self‐criticalness	0.31 ± 0.668	0.18 ± 0.573	0.11 ± 0.387	*H* = 2.681, *p* = 0.262	*F* = 0.917, *p* = 0.402
BDI‐9: Suicidal thoughts	0.08 ± 0.280	0.02 ± 0.140	0.05 ± 0.211	*H* = 2.937, *p* = 0.230	*F* = 1.123, *p* = 0.327
BDI‐10: Crying	0.08 ± 0.368	0.12 ± 0.553	0.14 ± 0.632	*H* = 0.024, *p* = 0.988	*F* = 0.039, *p* = 0.962
BDI‐11: Agitation	0.11 ± 0.319	0.12 ± 0.431	0.11 ± 0.321	*H* = 0.434, *p* = 0.805	*F* = 0.391, *p* = 0.677
BDI‐12: Loss of interest	0.22 ± 0.485	0.13 ± 0.337	0.14 ± 0.409	*H* = 1.297, *p* = 0.523	*F* = 0.358, *p* = 0.700
BDI‐13: Indecision	0.08 ± 0.280	0.09 ± 0.286	0.20 ± 0.462	*H* = 3.126, *p* = 0.209	*F* = 1.534, *p* = 0.218
BDI‐14: Worthlessness	0.00 ± 0.000	0.06 ± 0.238	0.07 ± 0.255	*H* = 2.388, *p* = 0.303	*F* = 1.246, *p* = 0.290
BDI‐15: Loss of energy	0.28 ± 0.615	0.31 ± 0.543	0.41 ± 0.542	*H* = 2.644, *p* = 0.267	*F* = 0.849, *p* = 0.429
BDI‐16: Change in sleeping pattern	0.25 ± 0.554	0.24 ± 0.532	0.27 ± 0.499	*H* = 0.459, *p* = 0.795	*F* = 0.734, *p* = 0.481
BDI‐17: Irritability	0.11 ± 0.319	0.04 ± 0.196	0.11 ± 0.493	*H* = 2.409, *p* = 0.300	*F* = 0.929, *p* = 0.397
BDI‐18: Change in appetite	0.19 ± 0.467	0.05 ± 0.218	0.11 ± 0.321	*H* = 5.068, *p* = 0.079	*F* = 2.446, *p* = 0.090
BDI‐19: Concentration difficulty	0.22 ± 0.485	0.17 ± 0.449	0.27 ± 0.585	*H* = 1.280, *p* = 0.527	*F* = 0.779, *p* = 0.460
BDI‐20: Tiredness or fatigue	0.31 ± 0.624	0.21 ± 0.432	0.25 ± 0.438	*H* = 0.703, *p* = 0.704	*F* = 0.563, *p* = 0.570
BDI‐Total	3.03 ± 4.712	2.55 ± 4.051	3.34 ± 5.447	*H* = 1.062, *p* = 0.588	*F* = 0.797, *p* = 0.452

*Note*: *H* indicates *H*‐value of Kruskal–Wallis *H*‐test.

### 3.4 IL‐1β SNPs rs1143630 GG, rs1143643 CC, and rs3136558 AA are associated with more severe depressive symptoms

The frequency of the minor rs1143630 T allele was 18.5% (Table 1). Since the number of individuals with the TT genotype of rs1143630 (*n* = 3) was relatively small, we combined individuals carrying the T allele rs1143630 (genotypes TG or TT) into one group for comparison with those possessing the GG genotype. We found no demographic characteristic differences between the two groups (Table 3). After controlling for confounding factors using Quade's test, individuals with the GG genotype of the rs1143630 SNP exhibited more severe symptoms for the “BDI‐13: indecision” item compared to those carrying the T allele of rs1143630 (*p* = 0.004; Table 3). Furthermore, based on Beck's classification of six depressive symptoms, the item can be categorized into dimension of emotion regulation.

**TABLE 3:** Clinical characteristics and BDI‐II scale results stratified by rs1143630 genotypes.

Variables	TG + TT (*n* = 64)	GG (*n* = 117)	Statistic, *p*‐value	Quade's test
Age (years)	60.61 ± 6.851	62.75 ± 7.707	*t* = 3.454, *p* = 0.065	
Male/female	27 (42.4%)/37 (57.8%)	58 (49.6%)/59 (50.4%)	χ^2^ = 0.906, *p* = 0.341	
Education (years)	11.65 ± 4.122	11.66 ± 3.623	*t* = 0.001, *p* = 0.987	
Marital status (single/married/divorced/widowed)	3/57/2/2	10/97/1/9	χ^2^ = 3.749, *p* = 0.290	
Sustained virologic response	28 (65.1%)	52 (73.2%)	χ^2^ = 0.844, *p* = 0.358	
FIB‐4 score	3.42 ± 9.962	2.25 ± 1.308	*F* = 1.575, *p* = 0.211	
HCV/HBV/both	38/21/5	66/46/5	χ^2^ = 1.546, *p* = 0.433	
HCV genotype (1a, 1b, 2, 1b/2, 1b/3, 2/4) (number)	0, 20, 18, 1, 0, 0	3, 21, 33, 2, 1, 1	χ^2^ = 5.475, *p* = 0.291	
BDI‐1: Sadness	0.00 ± 0.000	0.05 ± 0.258	*U* = 3584, *p* = 0.094	*F* = 3.634, *p* = 0.058
BDI‐2: Pessimism	0.13 ± 0.418	0.26 ± 0.635	*U* = 3418, *p* = 0.118	*F* = 2.587, *p* = 0.110
BDI‐3: Past failure	0.11 ± 0.441	0.18 ± 0.535	*U* = 3566, *p* = 0.295	*F* = 1.147, *p* = 0.286
BDI‐4: Loss of pleasure	0.03 ± 0.175	0.12 ± 0.351	*U* = 3444, *p* = 0.062	*F* = 3.549, *p* = 0.061
BDI‐5: Feeling of guilty	0.17 ± 0.380	0.14 ± 0.345	*U* = 3613, *p* = 0.527	*F* = 0.020, *p* = 0.888
BDI‐6: Feelings of being punished	0.16 ± 0.648	0.18 ± 0.638	*U* = 3633, *p* = 0.489	*F* = 0.575, *p* = 0.449
BDI‐7: Self‐dislike	0.03 ± 0.175	0.08 ± 0.326	*U* = 3635, *p* = 0.390	*F* = 0.902, *p* = 0.343
BDI‐8: Self criticalness	0.22 ± 0.629	0.17 ± 0.513	*U* = 3734, *p* = 0.955	*F* = 0.205, *p* = 0.651
BDI‐9: Suicidal thoughts	0.02 ± 0.125	0.05 ± 0.222	*U* = 3611, *p* = 0.236	*F* = 2.925, *p* = 0.089
BDI‐10: Crying	0.16 ± 0.648	0.09 ± 0.473	*U* = 3668, *p* = 0.547	*F* = 0.090, *p* = 0.765
BDI‐11: Agitation	0.08 ± 0.370	0.14 ± 0.392	*U* = 3483, *p* = 0.125	*F* = 2.805, *p* = 0.096
BDI‐12: Loss of interest	0.13 ± 0.333	0.16 ± 0.414	*U* = 3660, *p* = 0.677	*F* = 0.485, *p* = 0.487
BDI‐13: Indecision	0.03 ± 0.175	0.16 ± 0.393	*U* = 3284, *p* = 0.012*	*F* = 8.427, *p* = 0.004*
BDI‐14: Worthlessness	0.05 ± 0.213	0.05 ± 0.222	*U* = 3728, *p* = 0.897	*F* = 0.020, *p* = 0.887
BDI‐15: Loss of energy	0.28 ± 0.576	0.35 ± 0.546	*U* = 3429, *p* = 0.237	*F* = 1.366, *p* = 0.244
BDI‐16: Change in sleeping pattern	0.28 ± 0.629	0.23 ± 0.462	*U* = 3743, *p* = 0.995	*F* = 0.274, *p* = 0.601
BDI‐17: Irritability	0.05 ± 0.213	0.09 ± 0.361	*U* = 3662, *p* = 0.557	*F* = 0.615, *p* = 0.434
BDI‐18: Change in appetite	0.11 ± 0.362	0.09 ± 0.281	*U* = 3708, *p* = 0.828	*F* = 0.003, *p* = 0.959
BDI‐19: Concentration difficulty	0.11 ± 0.362	0.26 ± 0.544	*U* = 3321, *p* = 0.052	*F* = 4.350, *p* = 0.038
BDI‐20: Tiredness or fatigue	0.19 ± 0.531	0.26 ± 0.443	*U* = 3310, *p* = 0.073	*F* = 3.658, *p* = 0.057
BDI–Total	2.34 ± 4.183	3.11 ± 4.723	*U* = 3240, *p* = 0.114	*F* = 3.698, *p* = 0.056

* *p* < 0.0125. *U* indicates *U*‐value of Mann–Whitney *U*‐test.

The frequency of the minor rs1143643 C allele was 48.9% (Table 1). We observed no significant differences in demographic characteristics (Table 4). However, a statistically significant difference emerged in the “BDI‐11: agitation” item among the different genotype groups (*p* = 0.006; Table 4). To further investigate the specific genotype groups with significant differences in the “BDI‐11: agitation” item, a Quade post‐hoc analysis was conducted. This examination revealed that individuals with the CC genotype of rs1143643 exhibited more pronounced symptoms than those with the CT genotype (*p* = 0.001; Table 4). However, the post‐hoc analysis indicated no significant differences between individuals with the CC and TT genotypes, or between those with the CT and TT genotypes (*p* = 0.094 and *p* = 0.294, respectively). Employing the modified Beck's six‐symptom categorization, based on the BDI‐II, this item can be classified into a dimension reflecting high arousal type energy regulation.

**TABLE 4:** Clinical characteristics and BDI‐II scale results stratified by rs1143643 genotypes.

Variables	CC (*n* = 41)	CT (*n* = 95)	TT (*n* = 45)	Statistic, *p*‐value	Quade's test
Age (years)	60.37 ± 6.359	62.68 ± 7.776	62.02 ± 7.659	*F* = 1.387, *p* = 0.253	
Male/female	15 (36.6%)/26 (63.4%)	50 (52.6%)/45 (47.4%)	20 (44.4%)/25 (55.6%)	χ^2^ = 3.113, *p* = 0.211	
Education (years)	11.61 ± 4.224	11.71 ± 3.537	11.58 ± 4.014	*F* = 0.022, *p* = 0.978	
Marital status (single/married/divorced/widowed)	0/38/0/3	8/78/3/6	5/38/0/2	χ^2^ = 7.520, *p* = 0.275	
Sustained virologic response	19 (63.3%)	42 (79.2%)	19 (61.3%)	χ^2^ = 3.923, *p* = 0.141	
FIB‐4 score	4.21 ± 12.408	2.26 ± 1.343	2.10 ± 1.098	*F* = 1.778, *p* = 0.172	
HCV/HBV/both	25/11/5	51/42/2	28/14/3	χ^2^ = 8.613, *p* = 0.060	
HCV genotype (1a, 1b, 2, 1b/2, 1b/3, 2/4) (number)	0, 10, 15, 1, 0, 0	3, 21, 20, 1, 0, 1	0, 10, 16, 0, 1, 0	χ^2^ = 8.393, *p* = 0.586	
BDI‐1: Sadness	0.05 ± 0.218	0.02 ± 0.205	0.04 ± 0.208	*H* = 2.120, *p* = 0.346	*F* = 0.785, *p* = 0.458
BDI‐2: Pessimism	0.22 ± 0.525	0.19 ± 0.532	0.27 ± 0.688	*H* = 0.293, *p* = 0.864	*F* = 0.180, *p* = 0.835
BDI‐3: Past failure	0.32 ± 0.722	0.11 ± 0.399	0.11 ± 0.438	*H* = 3.983, *p* = 0.136	*F* = 1.790, *p* = 0.170
BDI‐4: Loss of pleasure	0.07 ± 0.264	0.07 ± 0.263	0.13 ± 0.405	*H* = 0.680, *p* = 0.712	*F* = 0.582, *p* = 0.560
BDI‐5: Feeling of guilty	0.15 ± 0.358	0.16 ± 0.367	0.13 ± 0.344	*H* = 0.148, *p* = 0.929	*F* = 0.359, *p* = 0.699
BDI‐6: Feelings of being punished	0.32 ± 0.820	0.08 ± 0.453	0.22 ± 0.765	*H* = 6.103, *p* = 0.047	*F* = 2.959, *p* = 0.054
BDI‐7: Self‐dislike	0.10 ± 0.300	0.03 ± 0.228	0.09 ± 0.358	*H* = 3.779, *p* = 0.151	*F* = 1.905, *p* = 0.152
BDI‐8: Self criticalness	0.24 ± 0.582	0.19 ± 0.607	0.13 ± 0.405	*H* = 1.374, *p* = 0.503	*F* = 0.408, *p* = 0.665
BDI‐9: Suicidal thoughts	0.07 ± 0.264	0.01 ± 0.103	0.07 ± 0.252	*H* = 4.262, *p* = 0.119	*F* = 1.374, *p* = 0.256
BDI‐10: Crying	0.10 ± 0.374	0.06 ± 0.433	0.24 ± 0.802	*H* = 3.510, *p* = 0.173	*F* = 1.685, *p* = 0.188
BDI‐11: Agitation	0.27 ± 0.549	0.06 ± 0.320	0.09 ± 0.288	*H* = 10.394, *p* = 0.006	CC vs. CT *F* = 10.989, *p* = 0.001*
					CT vs. TT *F* = 1.109, *p* = 0.294
					CC vs. TT *F* = 2.874, *p* = 0.094
BDI‐12: Loss of interest	0.27 ± 0.501	0.12 ± 0.322	0.11 ± 0.383	*H* = 5.151, *p* = 0.076	*F* = 2.016, *p* = 0.136
BDI‐13: Indecision	0.15 ± 0.358	0.09 ± 0.294	0.13 ± 0.405	*H* = 0.766, *p* = 0.682	*F* = 0.214, *p* = 0.807
BDI‐14: Worthlessness	0.07 ± 0.264	0.05 ± 0.224	0.02 ± 0.149	*H* = 1.208, *p* = 0.547	*F* = 0.669, *p* = 0.513
BDI‐15: Loss of energy	0.41 ± 0.631	0.27 ± 0.535	0.36 ± 0.529	*H* = 2.482, *p* = 0.289	*F* = 1.546, *p* = 0.216
BDI‐16: Change in sleeping pattern	0.24 ± 0.538	0.20 ± 0. 452	0.36 ± 0. 645	*H* = 2.312, *p* = 0.315	*F* = 1.298, *p* = 0.276
BDI‐17: Irritability	0.15 ± 0.358	0.02 ± 0.144	0.11 ± 0.487	*H* = 7.793, *p* = 0.020	*F* = 3.348, *p* = 0.037
BDI‐18: Change in appetite	0.17 ± 0.442	0.05 ± 0.224	0.11 ± 0.318	*H* = 3.572, *p* = 0.168	*F* = 1.596, *p* = 0.206
BDI‐19: Concentration difficulty	0.20 ± 0.459	0.17 ± 0.429	0.29 ± 0.626	*H* = 0.837, *p* = 0.658	*F* = 0.512, *p* = 0.600
BDI‐20 Tiredness or fatigue	0.37 ± 0.623	0.22 ± 0.442	0.16 ± 0.367	*H* = 3.502, *p* = 0.174	*F* = 1.578, *p* = 0.209
BDI‐Total	3.93 ± 5.561	2.21 ± 3.212	3.18 ± 5.674	*H* = 1.216, *p* = 0.544	*F* = 0.329, *p* = 0.720

* *p* < 0.0125. *H* indicates *H*‐value of Kruskal–Wallis *H*‐test.

The frequency of the minor rs3136558 G allele was 39.5% (Table 1). Since the number of individuals with the GG genotype of rs3136558 (*n* = 21) was <30, we combined those with the GG genotype with those with the AG genotype as one group, those carrying the G allele, to compare with those with the AA genotype rs3136558. Under similar demographic characteristics between the two groups (Table 5), Quade's test demonstrated that those with the AA genotype of rs3136558 were associated with more severe symptoms compared to those carrying the G allele in the item “BDI‐6: feeling of being punished” (*p* = 0.005; Table 5). Furthermore, this item can be classified into cognitive distortions based on Beck's six depressive symptoms.

**TABLE 5:** Clinical characteristics and BDI‐II scale results stratified by rs3136558 genotypes.

Variables	GG + AG (*n* = 122)	AA (*n* = 59)	Statistic, *p*‐value	Quade's test
Age (years)	62.53 ± 7.51	60.88 ± 7.29	*t* = 1.955, *p* = 0.164	
Male/female	63 (51.6%)/59 (48.4%)	22 (37.3%)/37 (62.7%)	χ^2^ = 3.288, *p* = 0.070	
Education (years)	11.82 ± 3.712	11.31 ± 3.971	*t* = 0.742, *p* = 0.390	
Marital status (single/married/divorced/widowed)	11/103/2/6	2/51/1/5	χ^2^ = 2.60, *p* = 0.457	
Sustained virologic response	54 (75.0%)	26 (61.9%)	χ^2^ = 2.173, *p* = 0.140	
FIB‐4 score	2.87 ± 7.268	2.22 ± 1.310	*F* = 0.463, *p* = 0.497	
HCV/HBV/both	65/50/7	39/17/3	χ^2^ = 2.751, *p* = 0.242	
HCV genotype (1a, 1b, 2, 1b/2, 1b/3, 2/4) (number)	3, 26, 28, 1, 1, 1	0, 15, 23, 1, 0, 0	χ^2^ = 3.920, *p* = 0.652	
BDI‐1: Sadness	0.01 ± 0.091	0.08 ± 0.337	*U* = 3384, *p* = 0.022	*F* = 4.012, *p* = 0.047
BDI‐2: Pessimism	0.20 ± 0.574	0.24 ± 0.567	*U* = 3425, *p* = 0.395	*F* = 0.847, *p* = 0.359
BDI‐3: Past failure	0.10 ± 0.394	0.27 ± 0.665	*U* = 3276, *p* = 0.053	*F* = 3.280, *p* = 0.072
BDI‐4: Loss of pleasure	0.08 ± 0.304	0.10 ± 0.305	*U* = 3502, *p* = 0.537	*F* = 0.160, *p* = 0.689
BDI‐5: Feeling of guilty	0.12 ± 0.330	0.20 ± 0.406	*U* = 3310, *p* = 0.156	*F* = 0.987 *p* = 0.322
BDI‐6: Feelings of being punished	0.09 ± 0.481	0.34 ± 0.863	*U* = 3139, *p* = 0.004*	*F* = 8.045, *p* = 0.005*
BDI‐7: Self‐dislike	0.06 ± 0.296	0.07 ± 0.254	*U* = 3507, *p* = 0.457	*F* = 0.396, *p* = 0.530
BDI‐8: Self criticalness	0.15 ± 0.525	0.27 ± 0.611	*U* = 3239, *p* = 0.050	*F* = 3.224, *p* = 0.074
BDI‐9: Suicidal thoughts	0.02 ± 0.156	0.07 ± 0.254	*U* = 3444, *p* = 0.159	*F* = 0.811, *p* = 0.369
BDI‐10: Crying	0.12 ± 0.597	0.10 ± 0.402	*U* = 3513, *p* = 0.487	*F* = 0.086, *p* = 0.769
BDI‐11: Agitation	0.08 ± 0.330	0.19 ± 0.473	*U* = 3287, *p* = 0.062	*F* = 2.640, *p* = 0.106
BDI‐12: Loss of interest	0.11 ± 0.345	0.22 ± 0.457	*U* = 3250, *p* = 0.077	*F* = 2.027, *p* = 0.156
BDI‐13: Indecision	0.11 ± 0.345	0.12 ± 0.326	*U* = 3559, *p* = 0.824	*F* = 0.013, *p* = 0.908
BDI‐14: Worthlessness	0.04 ± 0.199	0.07 ± 0.254	*U* = 3503, *p* = 0.438	*F* = 0.698, *p* = 0.405
BDI‐15: Loss of energy	0.31 ± 0.482	0.36 ± 0.689	*U* = 3532, *p* = 0.798	*F* = 0.003, *p* = 0.958
BDI‐16: Change in sleeping pattern	0.22 ± 0.506	0.31 ± 0.565	*U* = 3352, *p* = 0.291	*F* = 0.224, *p* = 0.636
BDI‐17: Irritability	0.07 ± 0.334	0.08 ± 0.281	*U* = 3474, *p* = 0.359	*F* = 0.640, *p* = 0.425
BDI‐18: Change in appetite	0.06 ± 0.234	0.17 ± 0.422	*U* = 3253, *p* = 0.033	*F* = 4.209, *p* = 0.042
BDI‐19: Concentration difficulty	0.20 ± 0.497	0.20 ± 0.484	*U* = 3584, *p* = 0.944	*F* = 0.009, *p* = 0.923
BDI‐20 Tiredness or fatigue	0.20 ± 0.419	0.32 ± 0.571	*U* = 3237, *p* = 0.128	*F* = 1.734, *p* = 0.190
BDI‐Total	2.39 ± 4.129	3.78 ± 5.210	*U* = 3035, *p* = 0.071	*F* = 2.027, *p* = 0.156

* *p* < 0.0125. *U* indicates *U*‐value of Mann–Whitney *U*‐test.

Lastly, we conducted a haplotype analysis to determine the combined effects of rs1143630 GG, rs1143643 CC, and rs3136558 on the depressive symptoms of the participants. However, due to a limited number of participants in some haplotype groups, our results did not find any significant differences in depressive symptoms across the different haplotype groups (Table 6).

**TABLE 6:** Haplotype analysis of IL1B the association with depressive symptoms in BDI scale.

rs1143630	rs1143643	rs3136558	Frequency	*p*‐value
T allele	C allele	G allele	0.215 (39/181)	BDI‐1: *H* = 10.126, *p* = 0.119 BDI‐2: *H* = 5.777, *p* = 0.449 BDI‐3: *H* = 9.107, *p* = 0.168 BDI‐4: *H* = 5.053, *p* = 0.537 BDI‐5: *H* = 5.890, *p* = 0.436 BDI‐6: *H* = 9.707, *p* = 0.138 BDI‐7: *H* = 4.897, *p* = 0.557 BDI‐8: *H* = 6.067, *p* = 0.416 BDI‐9: *H* = 5.020, *p* = 0.541 BDI‐10: *H* = 4.322, *p* = 0.633 BDI‐11: *H* = 13.512, *p* = 0.036 BDI‐12: *H* = 8.135, *p* = 0.228 BDI‐13: *H* = 9.701, *p* = 0.138 BDI‐14: *H* = 3.689, *p* = 0.719 BDI‐15: *H* = 5.026, *p* = 0.540 BDI‐16: *H* = 10.839, *p* = 0.094 BDI‐17: *H* = 5.446, *p* = 0.488 BDI‐18: *H* = 15.476, *p* = 0.027 BDI‐19: *H* = 6.016, *p* = 0.421 BDI‐20: *H* = 10.470, *p* = 0.106 BDI‐total: *H* = 6.054, *p* = 0.417
T allele	C allele	Non‐G allele	0.088 (16/181)	
T allele	Non‐C allele	G allele	0.049 (9/181)	
T allele	Non‐C allele	Non‐G allele	0.000 (0/181)	
Non‐T allele	C allele	G allele	0.265 (48/181)	
Non‐T allele	C allele	Non‐G allele	0.185 (33/181)	
Non‐T allele	Non‐C allele	G allele	0.143 (26/181)	
Non‐T allele	Non‐C allele	Non‐G allele	0.055 (10/181)	

## 4 DISCUSSION

This study identified three SNPs within the intronic region of the IL‐1β gene that influence the severity of depressive symptoms in individuals with chronic viral hepatitis. Previous studies have rarely examined the influence of the IL‐1β SNPs on depressive symptoms among individuals with chronic viral hepatitis, or the specific domain affected. Although these variations do not modify proteins directly, numerous studies have shown that variations in non‐coding regions can be phenotypically important by affecting the systems that regulate protein expression. These mechanisms include impacts on splicing, transcription, translation, RNA processing, and chromatin interactions.^24^

Our findings revealed that the rs1143630 GG and rs3136558 AA genotypes were associated with heightened severity in emotion regulation and cognitive distortions, respectively. Although previous studies have suggested the functional significance of these two SNPs (rs1143630 and rs3136558) in several diseases,^25^, ^26^ their potential effects on modulating mood symptoms remain unexplored. Additionally, we found the C allele of another SNP, rs1143643,^16^, ^17^ to be associated with more severe symptoms of energy regulation.

However, we observed no significant associations between the SNPs located in the promoter region, both rs16944 and rs1143627, and depressive symptoms, which contradicts previous assumptions of their relation to IL‐1β levels^27^ and the severity of depressive symptoms in Caucasian^10^, ^12^, ^13^ and Taiwanese^11^, ^14^ populations. The rs1143627 polymorphism, situated within the promoter sequence, has been postulated to enhance transcription factor binding and increase IL‐1β production due to the presence of the major A allele.^27^ Conversely, research conducted on the Finnish population identified an association between the minor G allele and elevated IL‐1β expression.^9^ Phenotypically, one study reported a significant association between the co‐occurrence of rs1143627 AA and rs16944 GG genotypes and the incidence of major recurrent depression.^13^

This lack of association in our study can be primarily attributed to two factors. First, individuals from diverse racial backgrounds may have unique mechanisms for modulating cytokine production. As a result, the increased IL‐1β expression associated with the rs1143627 A allele does not necessarily cause a corresponding elevation in IL‐1β levels within our study population. Second, the potential modulatory impact of viral hepatitis on the influence of the rs1143627 SNP could play a role in the development of depressive symptoms.

The rs1143630 is an intronic SNP of IL‐1β, situated near the 5′ region of the *IL1B* gene.^25^ Although no published research has specifically investigated the relationship between rs1143630 and depressive disorders or provided definitive evidence concerning the IL‐1β levels associated with SNP functionality, some studies have suggested that rs1143630 may cause susceptibility to certain diseases.^25^, ^26^ Phenotypically, one study found that individuals carrying the rs1143630 G allele were predisposed to osteoporosis in the Chinese population,^26^ potentially due to the more potent effect of IL‐1β on osteoclast precursor differentiation into mature osteoclasts. Moreover, the rs1143630 T allele has been identified as exerting a protective effect against cervical cancer susceptibility,^25^ possibly attributable to the reduced pro‐inflammatory nature of IL‐1β, which reduces tumor invasion. In alignment with previous studies in the Chinese population,^25^, ^26^ our results suggest that the GG genotype of rs1143630 is associated with more severe depressive symptoms and elevated IL‐1β expression, which is compatible with one previous in silico study.^25^

Rs3136558 is an intronic SNP of IL‐1β located in the 5′ region of the *IL1B* gene. Several studies have demonstrated the correlation between the IL‐1β SNP and susceptibility to certain diseases.^25^, ^28^ For example, the Chinese study showing the protective effect of the rs1143630 T allele also found the rs3136558 G allele to exhibit a protective effect against cervical cancer susceptibility.^25^ However, there has been no reported association between rs3136558 and depressive disorder. Few studies have reported the differences in IL‐1β levels between individuals with different rs3136558 alleles, with one study suggesting that this SNP may be involved in the regulation of transcription.^25^ In summary, our data corroborate the research finding conducted within the Chinese population,^25^ suggesting that the rs3136558 AA genotype is associated with higher IL‐1β levels and more severe depressive symptoms.

The rs1143643 variant, an intronic polymorphism situated near the 3′ terminus of the IL‐1β gene, has yet to have its functional implications definitively elucidated. A prior investigation employing in silico techniques proposed a potential role of rs1143643 in gene transcription.^25^ Our findings revealed a significant association between the CC genotype of rs1143643 and the increased severity of depressive symptoms. In line with our results, previous research has identified the CC genotype of rs1143643 as a predictor of suboptimal treatment outcomes in antidepressant therapies, alongside diminished responses within the amygdala and anterior cingulate cortex during emotional stimulation.^17^

There is substantial biological evidence supporting our findings regarding the adverse impact of elevated IL‐1β levels on the development of depressive symptoms. IL‐1β is expressed in glial cells within the cerebral cortex and hypothalamus, as well as neurons in the hippocampus, whereas its receptors are predominantly located in the hippocampus, hypothalamus, anterior cingulate cortex, and dentate gyrus.^29^, ^30^ The unique distribution of cytokines and their receptors in these specific brain regions contributes to their distinct effects. For example, IL‐1β influences tryptophan metabolism in human hippocampal progenitor cells by inducing the upregulation of indoleamine‐2,3‐dioxygenase (IDO) enzyme, thereby reducing tryptophan availability for serotonin synthesis.^31^ In addition, IL‐1β has been implicated in impairing striatal dopamine neurotransmission.^32^ These findings shed light on the mechanisms through which IL‐1β may disrupt emotion regulation, as observed in our study.

There is also a compelling biological basis for the association between cognitive distortion and elevated IL‐1β levels. For instance, when IL‐1β levels produced by microglial cells exceed physiological thresholds, they may adversely impact neural synaptic plasticity and compromise hippocampal learning and memory processes.^29^ Furthermore, IL‐1β interferes with brain‐derived neurotrophic factor signaling pathways,^33^ disrupts glutamatergic synaptic transmission in hippocampal pyramidal neurons,^34^ and accelerates norepinephrine and dopamine turnover.^35^

In addition, elevated IL‐1β levels have been implicated in dysregulated energy regulation. IL‐1β has been demonstrated to trigger hyperactivation of the hypothalamic–pituitary–adrenal axis.^36^, ^37^ Hypothalamic–pituitary–adrenal axis hyperactivation give rise to heightened energy regulation, such as agitation.^38^ Moreover, sustained elevations in downstream glucocorticoid levels can contribute to the atrophy of hippocampal pyramidal neurons and the medial prefrontal cortex, potentially resulting in depression with cognitive symptoms.^39^, ^40^

Despite its merits, the current study acknowledges several potential limitations. Firstly, the sample size restricted our ability to categorize participants based on their HBV or HCV status, thus hindering the investigation of IL‐1β SNPs’ distinct impacts on either HBV or HCV. Additionally, our study cohort included individuals with untreated chronic HCV as well as those who achieved SVR post‐treatment. This heterogeneity in HCV infection status may have influenced participants’ depressive symptoms. Nonetheless, χ^2^ test results confirmed an equal distribution of patients achieving SVR across each analyzed IL‐1β SNP group, suggesting that this heterogeneity does not compromise the study's validity. Lastly, in vivo IL‐1β levels were not assessed in the current study. Future research incorporating laboratory data, such as IL‐1β concentrations in blood or cerebrospinal fluid, would more effectively clarify the cytokine's role in developing depressive symptoms. While validating the results in an independent cohort with a larger number of sample size would strengthen our conclusions, it remains beyond the scope of this study. Subsequent research with a more extensive cohort can offer further validation. To conclude, we have comprehensively examined the relationship between depressive symptoms and IL‐1β SNPs in individuals with chronic viral hepatitis. Our analysis revealed a significant association between three intronic IL‐1β SNPs (rs1143630, rs1143643, and rs2853550) and the manifestation of depressive symptoms. Moreover, we have delineated three distinct dimensions of depressive symptoms influenced by these genetic variants, including disturbances in emotion regulation, cognitive distortions, and energy regulation. By establishing this correlation, we may optimize personalized medicine to prevent depression in chronic hepatitis patients proactively.

## CONFLICT OF INTEREST STATEMENT

The authors declare no conflict of interest.

## Supporting information


**DATA S1:** Supplementary Information
