# Supplementary material for: Impact of interleukin‐1β single nucleotide polymorphisms and depressive symptoms in individuals with chronic viral hepatitis
Source: Kaohsiung J Med Sci. 2023 Nov 8;40(1):94–104. doi: 10.1002/kjm2.12776 (PMC11895661; doi:10.1002/kjm2.12776)
Supplement: Supplementary file 1 — DATA S1: Supplementary Information [file KJM2-40-94-s001.docx]

**Supplementary Table 1. The treatment of chronic HCV patients recruited in the study.**

| Treatment regimen | SVR (n = 80) | Non-SVR (n = 34) |
| --- | --- | --- |
| Treatment naïve | - | n =33 (97%) |
| Peginterferon | n =44 (55%) | n =1　(3%) |
| Direct-acting antiviral agents (DAAs) | n =33 (41.3%) | - |
| Both DAAs and Peginterferon | n =3 (3.7%) | - |

**Supplementary Table 2. HCV genotype (GT) in the study.**

| HCV genotype (GT) | Number (percentage) |
| --- | --- |
| Missed data | n =15 (13.2%) |
| GT 1a | n =3 (3.0%) |
| GT 1b | n =41 (41.4%) |
| GT 2 | n =51 (51.5%) |
| mixed infection |  |
| GT 1b/2 | n =2 (2.0%) |
| GT 1b/3 | n =1 (1.0%) |
| GT 2/4 | n =1 (1.0%) |

**Supplementary Table 3. The liver fibrosis status of the recruited patients.**

| Instrument for assessing liver fibrosis status | Metavir score | FIB-4 score |
| --- | --- | --- |
| Available data / total patient number | 33 (18.2%) / 181 | 181 (100%) / 181 |
| F0 / F1 / F2 / F3 / F4 (number) | 0 / 9 / 4 / 1 / 19 | - |
| mean ± SD | - | 2.66 ± 6.01 |
| Range | - | 0.80-81.25 |

**Supplementary Table 4. Relationship between depressive symptoms based on BDI-II scale and HCV genotypes.**

| Variables | 1a (n = 3) | 1b (n = 41) | 2 (n = 51) | 1b/2 (n = 2) | 1b/3 (n = 1) | 2/4 (n = 1) | Statistic, *p*-value |
| --- | --- | --- | --- | --- | --- | --- | --- |
| BDI-1: Sadness | 0.00 ±0.000 | 0.05 ±0.218 | 0.02 ±0.140 | 0.00 ±0.000 | 0.00 ±0.000 | 0.00 ±0.000 | H=0.885, *p* =0.971 |
| BDI -2: Pessimism | 0.00 ±0.000 | 0.27 ±0.549 | 0.20 ±0.693 | 0.00 ±0.000 | 0.00 ±0.000 | 0.00 ±0.000 | H=4.278, *p* =0.510 |
| BDI -3: Past failure | 0.00 ±0.000 | 0.12 ±0.400 | 0.16 ±0.505 | 0.00 ±0.000 | 0.00 ±0.000 | 0.00 ±0.000 | H=0.747, *p* =0.980 |
| BDI -4: Loss of pleasure | 0.33 ±0.577 | 0.05 ±0.218 | 0.08 ±0.337 | 0.00 ±0.000 | 0.00 ±0.000 | 0.00 ±0.000 | H=4.147, *p* =0.528 |
| BDI -5: Feeling of guilty | 0.00 ±0.000 | 0.22 ±0.419 | 0.14 ±0.348 | 0.00 ±0.000 | 0.00 ±0.000 | 0.00 ±0.000 | H=2.561, *p* =0.767 |
| BDI -6: Feelings of being punished | 0.00 ±0.000 | 0.24 ±0.799 | 0.16 ±0.612 | 0.00 ±0.000 | 0.00 ±0.000 | 0.00 ±0.000 | H=0.787, *p* =0.978 |
| BDI -7: Self-dislike | 0.00 ±0.000 | 0.05 ±0.312 | 0.10 ±0.361 | 0.00 ±0.000 | 0.00 ±0.000 | 0.00 ±0.000 | H=1.699, *p* =0.889 |
| BDI -8: Self criticalness | 0.00 ±0.000 | 0.20 ±0.511 | 0.29 ±0.729 | 0.00 ±0.000 | 0.00 ±0.000 | 0.00 ±0.000 | H=1.296, *p* =0.935 |
| BDI -9: Suicidal thoughts | 0.00 ±0.000 | 0.10 ±0.300 | 0.04 ±0.196 | 0.00 ±0.000 | 0.00 ±0.000 | 0.00 ±0.000 | H=1.826, *p* =0.873 |
| BDI -10: Crying | 0.00 ±0.000 | 0.24 ±0.767 | 0.10 ±0.458 | 0.00 ±0.000 | 0.00 ±0.000 | 0.00 ±0.000 | H=1.153, *p* =0.949 |
| BDI -11: Agitation | 0.00 ±0.000 | 0.07 ±0.264 | 0.14 ±0.448 | 0.00 ±0.000 | 0.00 ±0.000 | 0.00 ±0.000 | H=0.884, *p*=0.971 |
| BDI -12: Loss of interest | 0.00 ±0.000 | 0.20 ±0.459 | 0.14 ±0.401 | 0.00 ±0.000 | 0.00 ±0.000 | 0.00 ±0.000 | H=1.669, *p* =0.893 |
| BDI -13: Indecision | 0.00 ±0.000 | 0.12 ±0.331 | 0.10 ±0.300 | 0.00 ±0.000 | 0.00 ±0.000 | 0.00 ±0.000 | H=0.979, *p* =0.964 |
| BDI -14: Worthlessness | 0.00 ±0.000 | 0.05 ±0.218 | 0.10 ±0.196 | 0.00 ±0.000 | 0.00 ±0.000 | 0.00 ±0.000 | H=0.367, *p* =0.996 |
| BDI -15: Loss of energy | 0.00 ±0.000 | 0.39 ±0.628 | 0.27 ±0.568 | 0.00 ±0.000 | 1.00 ±0.000 | 1.00 ±0.000 | H=7.867, *p* =0.164 |
| BDI -16: Change in sleeping pattern | 0.00 ±0.000 | 0.32 ±0.687 | 0.25 ±0.483 | 0.50 ±0.707 | 0.00 ±0.000 | 0.00 ±0.000 | H=2.177, *p* =0.824 |
| BDI -17: Irritability | 0.00 ±0.000 | 0.05 ±0.218 | 0.06 ±0.238 | 0.00 ±0.000 | 0.00 ±0.000 | 0.00 ±0.000 | H=0.444, *p* =0.994 |
| BDI -18: Change in appetite | 0.00 ±0.000 | 0.12 ±0.400 | 0.10 ±0.300 | 0.00 ±0.000 | 0.00 ±0.000 | 0.00 ±0.000 | H=0.746, *p* =0.980 |
| BDI -19: Concentration difficulty | 0.00 ±0.000 | 0.24 ±0.538 | 0.18 ±0.518 | 0.50 ±0.707 | 0.00 ±0.000 | 1.00 ±0.000 | H=7.586, *p* =0.181 |
| BDI -20 Tiredness or fatigue | 0.00 ±0.000 | 0.29 ±0.602 | 0.16 ±0.367 | 0.00 ±0.000 | 0.00 ±0.000 | 1.00 ±0.000 | H=6.666, *p* =0.247 |
| BDI -Total | 0.33 ±0.577 | 3.43 ±4.680 | 2.70 ±4.583 | 1.00 ±0.000 | 1.00 ±0.000 | 3.00 ±0.000 | H=2.753, *p* =0.738 |

**Supplementary Table 5. Allele frequency of IL-1B in our study and other population**

| SNP ID | Study | Population | Sample size | Minor / Major allele | MAF | *p*-value between different population |
| --- | --- | --- | --- | --- | --- | --- |
| rs16944 | Our study | Taiwanese | 181 | A/G | 46.7% | χ2=0.6868 (p=0.407) |
|  | Liu et al. ^1^ | Chinese | 1266 | A/G | 49.0% |  |
| rs1143627 | Our study | Taiwanese | 181 | G/A | 47.8% | χ2=0.0098 (p=0.921) |
|  | Liu et al. ^1^ | Chinese | 1266 | G/A | 47.5% |  |
| rs1143630 | Our study | Taiwanese | 181 | T/G | 18.5% | χ2=0.6993 (p=0.403) |
|  | He et al. ^2^ | Chinese | 1011 | T/G | 16.7% |  |
| rs1143636 | Our study | Taiwanese | 181 | G/A | 0% | - |
|  | dbSNP^3^ | East Asian | 132 | G/A | 0% |  |
| rs1143642 | Our study | Taiwanese | 181 | A/G | 0% | - |
|  | dbSNP | East Asian | 88 | A/G | 0% |  |
| rs1143643 | Our study | Taiwanese | 181 | C/T | 48.9% | χ2= 2.3185 (p=0.128) |
|  | dbSNP | East Asian | 86 | C/T | 42.0% |  |
| rs2853550 | Our study | Taiwanese | 181 | A/G | 7.7% | χ2= 0.3448 (p=0.557) |
|  | dbSNP | East Asian | 492 | A/G | 8.7% |  |
| rs3136558 | Our study | Taiwanese | 181 | G/A | 39.5% | χ2=0.8228 (p=0.364) |
|  | dbSNP | East Asian | 552 | G/A | 42.2% |  |
| rs3917350 | Our study | Taiwanese | 181 | G/A | 0% | - |
|  | dbSNP | East Asian | 86 | G/A | 0% |  |
| rs140794289 | Our study | Taiwanese | 181 | A/G | 2.8% | χ2= 2.0733 (p=0.150) |
|  | dbSNP | East Asian | 86 | A/G | 5% |  |

The database of single nucleotide polymorphisms (dbSNP); National Institutes of Health (NIH)

[1] Liu J, Zhai X, Jin G, Hu Z, Wang S, Wang X, et al. Functional variants in the promoter of interleukin-1β are associated with an increased risk of breast cancer: A case-control analysis in a Chinese population. 2006;118(10):2554-8.

[2] He Z, Sun Y, Wu J, Xiong Z, Zhang S, Liu J, et al. Evaluation of genetic variants in IL-1B and its interaction with the predisposition of osteoporosis in the northwestern Chinese Han population. The journal of gene medicine 2020;22(10):e3214.

[3] Sherry ST, Ward MH, Kholodov M, Baker J, Phan L, Smigielski EM, et al. dbSNP: the NCBI database of genetic variation. Nucleic acids research 2001;29(1):308-11.
